# Clinical and genetic study of a Chinese family affected by both amyotrophic lateral sclerosis and autosomal dominant polycystic kidney disease

**DOI:** 10.3389/fneur.2022.1004909

**Published:** 2022-10-20

**Authors:** Shirong Li, Junyu Lin, Chunyu Li, Yongping Chen, Bei Cao, Tianmi Yang, Qianqian Wei, Bi Zhao, Xueping Chen, Huifang Shang

**Affiliations:** ^1^Department of Neurology, Laboratory of Neurodegenerative Disorders, Rare Diseases Center, National Clinical Research Center for Geriatrics, West China Hospital, Sichuan University, Chengdu, China; ^2^Department of Neurology, Guizhou Provincial People's Hospital, Guiyang, China

**Keywords:** amyotrophic lateral sclerosis, *SOD1*, autosomal dominant polycystic kidney disease (ADPKD), *PKD1*, Chinese

## Abstract

Amyotrophic lateral sclerosis (ALS) is a progressive neurodegenerative disorder characterized by loss of the upper and lower motor neurons from the motor cortex, brainstem, and spinal cord. Most ALS cases are sporadic, with 5–10% having a positive family history. Autosomal dominant polycystic kidney disease (ADPKD) is a heritable renal disease that eventually results in end-stage kidney disease. *PKD1* is the most prevalent causative gene for ADPKD, accounting for ~85% of cases. Both diseases are currently considered untreatable. In this study, we report a large family that includes 10 patients with ALS phenotype, 3 asymptomatic *SOD1*-H47R carriers, and 6 with the ADPKD phenotype. Using whole exome sequencing, we found a novel likely pathogenic variant (p.R2787P) in *PKD1* among patients with ADPKD, and a pathogenic variant (p.H47R) in *SOD1* among patients with ALS. This study highlights the possibility that two different autosomal dominantly inherited diseases can co-exist independently within the same family. Phenotype—genotype correlations among these patients are also described. This research contributes novel phenotype and genotype characteristics of ALS with *SOD1* mutations and ADPKD with *PKD1* mutations.

## Introduction

Amyotrophic lateral sclerosis (ALS) is a neurodegenerative disorder mainly characterized by degeneration of upper and lower motor neurons, which causes death at a median of 3 years after onset (1). A recent study determined an average global incidence of 1.59 and a prevalence of 4.42 per 100,000 individuals (2). Family ALS (fALS) is mainly caused by mutations of four genes: *C9ORF72, SOD1, FUS*, and *TARDBP*. Variants of the copper zinc superoxide dismutase 1 (*SOD1*) gene are responsible for 10–20% of fALS cases (3).

Autosomal dominant polycystic kidney disease (ADPKD) is the most common hereditary renal disease, with an estimated prevalence of 1 in 2,000–4,000 individuals (4–6). About half of patients with ADPKD develop end-stage kidney disease (ESKD) by age 60 (7). ADPKD presents structural and functional kidney defects and various accompanying extrarenal complications. Polycystic kidney disease (PDK) 1 (*PKD1*) and 2 (*PKD2*) have been identified as the genes related to ADPKD (8).

To date, no cases of co-occurrence of these two genetic diseases, PKD and ALS, within a single family have been reported. Herein, we report a large family in which *SOD1*-ALS and *PKD1*-ADPKD occurred independently.

## Materials and methods

### Family members

**Figure 1:**
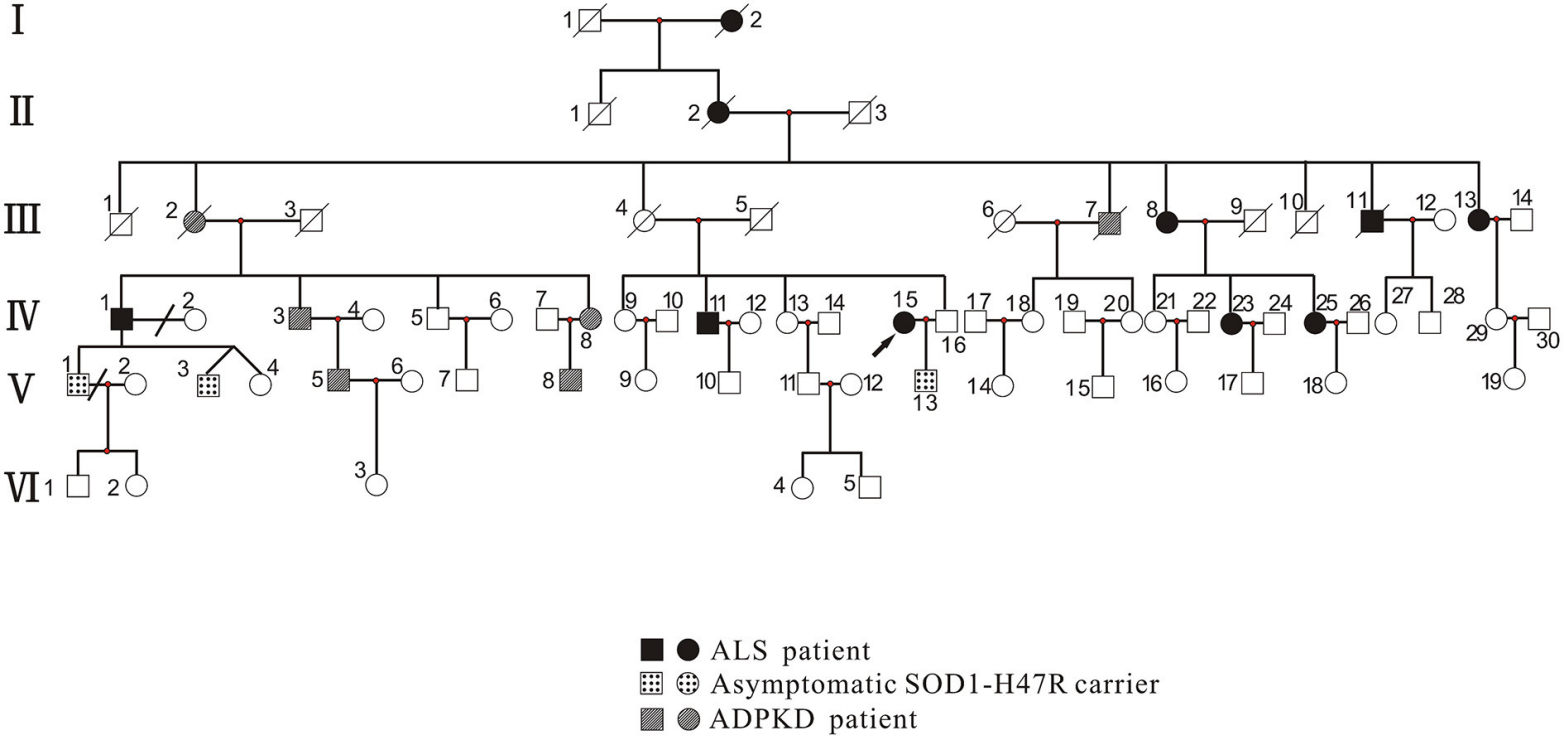
Pedigree of family with ALS and ADPKD. Patients with ALS included those with the ALS phenotype (III-13, IV-1, IV-11, IV-15, IV-23, and IV-25) who carried the SOD1 p.H47R mutation (note that I-2, II-2, III-8, and III-11 did not undergo genetic testing). Asymptomatic SOD1-H47R carriers were those who had no ALS phenotype but carried the SOD1 p.H47R mutation (V-1, V-3, and V-13). Patients with ADPKD had the ADPKD phenotype (IV-3, IV-8, V-5, and V-8) and carried the PKD1 p.R2787P variant (note that III-2 and III-7 did not undergo genetic testing). Circles indicate women. Squares indicate men. Arrow indicates proband. Diagonal lines indicate deceased individuals.

Proband and family members, who were from the Han Chinese population, were recruited by the Department of Neurology, West China Hospital of Sichuan University. Clinical investigations indicated that there was a total of 73 family members, among which 10 patients with ALS phenotype, 3 asymptomatic *SOD1*-H47R carriers, and 6 with the ADPKD phenotype (Figure 1). Patients with ALS were examined by at least two senior neurologists and diagnosed according to the revised EI Escorial criteria (9). Deceased patients had presented with the ALS or ADPKD phenotype before death.

The ADPKD diagnostic criteria were: aged < 30 years with >2 renal cysts (on one side or 1 on each side); age 30–59 years with ≥2 cysts (on each side); or aged ≥60 years with ≥4 cysts (on each side). However, the diagnostic criteria can be modified if the patient has a family history, extrarenal manifestations, or positive genetic tests for diseases such as polycystic liver, intracranial aneurysms, or cardiac valvular abnormalities (10). The following clinical data were collected: sex, age, education, height, weight, age of onset, site of symptoms onset, presence of respiratory failure, and wheelchair dependency. Body mass index (BMI) was calculated as body weight (kg) divided by height squared (m^2^). Survival time was defined as the duration from disease onset to death. Detailed neurological examinations were conducted to assess upper and lower motor neuron signs. Disease severity was evaluated using the ALS functional rating scale-revised (ALSFRS-R). Frontal lobe function was assessed using the Frontal Assessment Battery (FAB) scale. Cognitive impairment was assessed using the Montreal Cognitive Assessment (MoCA) scale. We also collected blood and cerebrospinal fluid (CSF) samples of the proband members. Electromyography (EMG), abdominal Doppler ultrasound (DU), and magnetic resonance imaging (MRI) of the head or abdomen were also conducted on patients. This research was approved by the Ethics Committee of the West China Hospital, Sichuan University.

### Whole exome sequencing and multiplex ligation-dependent probe amplification

A total of 19 DNA samples were available. Genomic DNA from blood samples was extracted using standard procedures (QIAGEN, Valencia, CA). Using a GenCap capture kit (MyGenostics GenCap Enrichment Technologies, Beijing, China). Enrichment libraries were sequenced using a DNBSEQ (DNBSEQ-T7) (MGI, Shenzhen, China) sequence for paired-end reads of 150 bp. After sequencing, raw data were saved in FASTQ format. Quality control filters were applied to remove low-quality reads. Reads were aligned to the human reference genome (UCSC Genome Browser build hg19) using Burrows-Wheeler Aligner20. Single-nucleotide polymorphism (SNP) and insertion/deletion variants were detected by GATK HaplotypeCaller. Data were then transformed to variant call format and variants further annotated by ANNOVAR and associated with multiple databases, including 1000 Genomes, ESP6500, dbSNP, and ExAC, and predicted by SIFT, PolyPhen-2, Mutation Taster, and GERP++. Pathogenicity of variation loci was also analyzed according to the American College of Medical Genetics and Genomics (ACMG) genetic variation classification criteria and guidelines (11, 12). Sanger sequencing was used to confirm identified mutations. MPLA was also conducted to exclude spinal muscular atrophy (SMA).

## Results

### Clinical and genetic features of the 10 patients with ALS and 3 asymptomatic *SOD1*-H47R carriers

**Figure 2:**
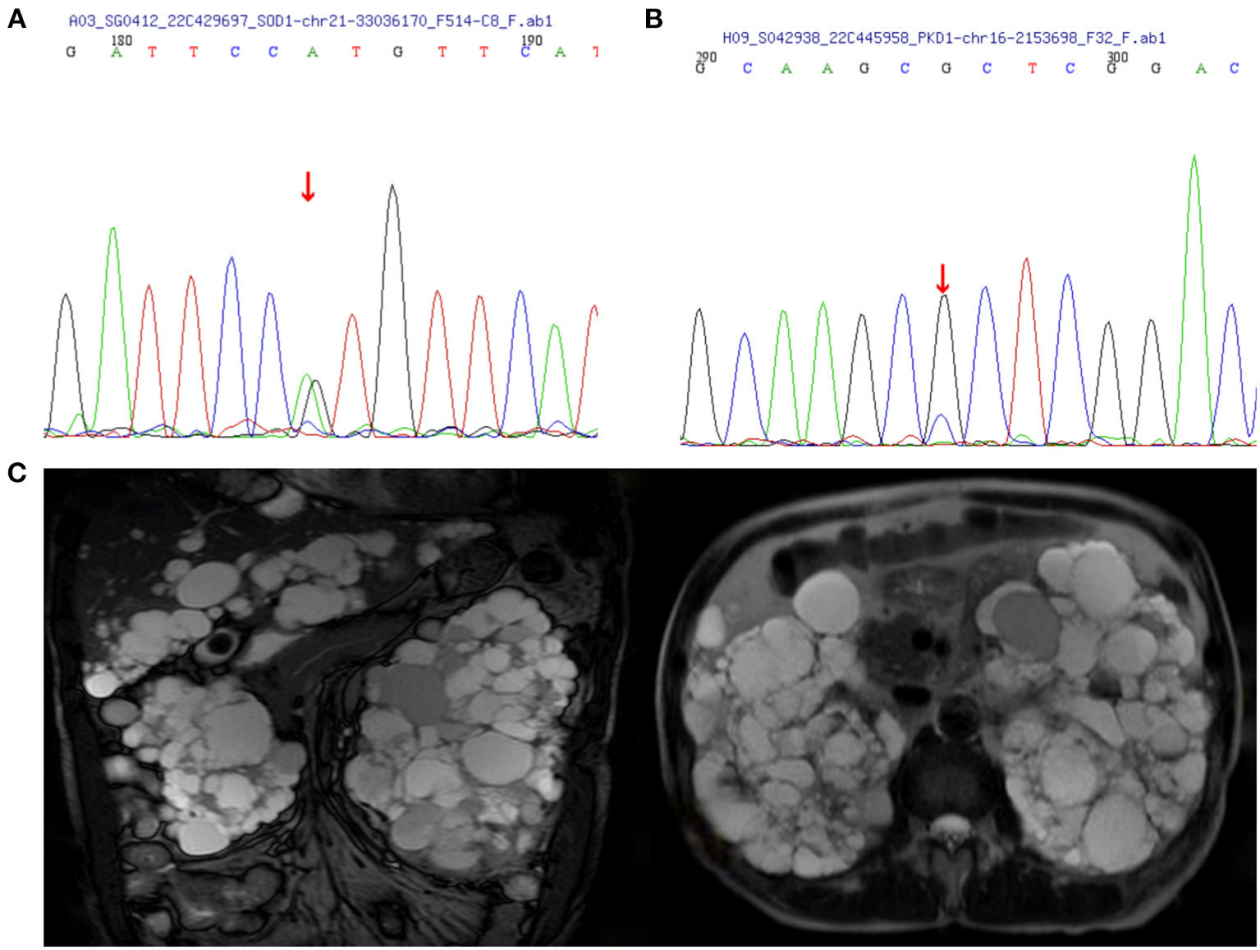
Images of polycystic kidney disease and sanger sequence. **(A)** SOD1:c.140(exon2)A>G. **(B)** PKD1:c.8360(exon23)G>C. **(C)** MRI images of the case IV-3 associated with polycystic liver disease and polycystic kidney disease.

The proband (Figure 1, IV-15) was a 57-year-old female with an 11 year history of progressive muscle weakness. The patient first noticed right lower limb weakness at age 46, followed by weakness and atrophy of all four limbs with proximal to distal progression. Neurological examination revealed severe muscle weakness in upper [Medical Research Council (MRC) 3/5] and lower (MRC 2/5) limbs, marked atrophy of all four limbs, hyporeflexia of all four limbs, and decreased muscle tone. No other abnormal neurological signs were found. She became wheelchair-dependent 6 years after onset. At diagnosis, her ALSFRS-R score was 27, the MoCA score was 27 (with 6 years of education), and she had a BMI of 24.0 kg/m^2^. Neurofilament light chain (NFL) in CSF was significantly elevated (1,349.27 pg/ml, normal: < 830.00 pg/ml). EMG revealed acute and chronic denervation in muscles of all limbs and rectus abdominis. Head MRI and abdominal DU showed no abnormalities. MLPA detected no abnormality in *SMN1/SMN2* genes. WES revealed a pathogenic (PS3+PS4+PM1+PM2_Supporting+PM5) variant (c.140A>G p.H47R) in the *SOD1* gene (Figure 2A). Verified *SOD1* genes of other family members are shown in Supplementary Table 1. Unfortunately, the proband's parents were deceased and their clinical data and genetic evaluations were unavailable.

Genetic testing revealed a c.140A>G (p.H47R) variant in the *SOD1* gene in members of III-13, IV-1, IV-11, IV-23, IV-25, V-1, V-3, and V-13, in addition to the IV-15 proband (Supplementary Table 1). At enrollment, 3 family members, including V-1 (age 40 years), V-3 (age 19 years), and V-13 (age 30 years) had no symptoms of muscle weakness or atrophy or abnormal neurological signs. Among all affected members, I-2, II-2, III-8, and III-11 did not receive genetic screening for *SOD1* as they were deceased, or DNA samples were unavailable. Detailed clinical characteristics are shown in Table 1. All patients, including I-2, II-2, III-8, III-11, III-13, IV-1, IV-11, IV-23, and IV-25, in addition to the proband, developed lower limb weakness as their initial symptom, which progressed to other limbs, with no bulbar onset. Aside from IV-11, those who had 2 years of education had lower MoCA and FAB scores (17 and 14, respectively), and no patient had cognitive impairment. I-2 and III-11 had long survival times (20 and 30 years, respectively), though II-2 had asphyxia during the final two survival years.

**Table 1:** Clinical and genetic features of 10 patients with ALS and 3 asymptomatic SOD1-H47R carriers.

**Patient**	**Sex**	**Age (y)**	**Education (y)**	**AAO** **(y)**	**Survival time (y)**	**Site of onset**	**UL**	**LL**	**BI**	**Tendon reflex**	**Muscle tone**	**Barbinski sign**	**Respirat ory failure**	**Phenotype**	**WD** **(y)**	**ALSFRS-R scores**	**BMI (kg/m^2^)**	**MoCA scores**	**SOD1**
I-2	F	NA	NA	60	20 (D)	LL	+	+	–	NA	NA	NA	+	LMN	NA	NA	NA	NA	NA
II-2	F	NA	NA	57	2 (D)	LL	–	+	–	NA	NA	NA	+	LMN	NA	NA	NA	NA	NA
III-8	F	NA	NA	40	43 (A)	LL	+	+	–	NA	NA	NA	–	LMN	36	NA	NA	NA	NA
III-11	M	NA	NA	36	30 (D)	LL	+	+	–	NA	NA	NA	+	LMN	14	NA	NA	NA	NA
III-13	F	69	9	39	30 (A)	LL	+	+	–	Hypo	DE	–	–	LMN	8	23	20.0	22	p.H47R
IV-1	M	71	12	60	11 (A)	LL	–	+	–	Hypo	DE	–	–	LMN	–	42	26.3	22	p.H47R
IV-11	M	64	2	58	6 (A)	LL	–	+	–	Hypo	DE	–	–	LMN	–	42	22.5	17	p.H47R
IV-15	F	57	6	46	11 (A)	LL	+	+	–	Hypo	DE	–	–	LMN	6	27	24.0	27	p.H47R
IV-23	F	56	15	44	12 (A)	LL	–	+	–	Hypo	DE	–	–	LMN	–	46	22.2	27	p.H47R
IV-25	F	54	12	54	1/12 (A)	LL	–	+	–	Hyper	IN	+	–	LMN+ UMN	–	47	24.7	28	p.H47R
V-1 AS	M	40	15	–	–	–	–	–	–	–	–	–	–	–	–	–	22.0	28	p.H47R
V-3 AS	M	19	12	–	–	–	–	–	–	–	–	–	–	–	–	–	20.0	28	p.H47R
V-13AS	M	30	9	–	–	–	–	–	–	–	–	–	–	–	–	–	25.0	29	p.H47R

AS, asymptomatic SOD1-H47R carrier; Sex: F, female; M, male; y, year; NA, information not available; AAO, age of onset; D, dead; A, alive; LL, lower limb involvement; UL, upper limb involvement; BI, bulbar involvement; +, presence; –, absence; Hypo, hyporeflexia; Hyper, hyperreflexia; DE, decreased muscle tone; IN, increased muscle tone; LMN, lower motor neuron; UMN, upper motor neuron; WD, duration from onset to wheelchair; ALSFRS-R, ALS functional rating scale-revised; BMI, body mass index; MoCA, montreal cognitive assessment; p.H47R, carried the SOD1 p.H47R mutation by genetic testing.

Neurological examination of all affected and available members, except for IV-25 showed lower motor neuron involvement. IV-25 had mild lower limb weakness and predominate upper motor neuron affected signs including increased muscle tone, hyperreflexia, and positive Hoffmann and Babinski signs. All patient EMGs showed neurogenic changes in limb and paraspinal muscles.

### Clinical and genetic features of 6 patients with ADPKD

Within this family, case IV-3 was a 70-year-old man who developed gross hematuria and hypertension, and his abdominal MRI (Figure 2C) showed multiple liver cysts and renal cysts when he was age 40 years. He was diagnosed with PKD and later developed ESKD at age 60 when he began hemodialysis therapy. His most recent (April 18, 2022) blood biochemical indices were urea 17.4 mmo/l, creatinine 1,176 umol/l and glomerular filtration rate (GFR) < 15 ml/min. WES revealed a novel heterozygous missense variant (c.8360G>C p.R2787P) on exon 23 of the *PKD1* gene (Figure 2B). The validated *PKD1* genes of other family members are shown in Supplementary Table 1. The integrative genomics viewer (IGV) screenshot shows the number of reads supporting each allele (Supplementary Figure 4). The sequencing depth of chr16:2153698 (PKD1 p.R2787P) is 438 and the “G” variant was 208. This variant is located in the mutation hot spot area (Supplementary Figures 3A,B) and was absent from both the 1000 Genomes Project and the Human Genetic Variation Database. It was predicted as harmful by multiple software analyses (SIFT, PolyPhen-2, Mutation Taster, and GERP++). The homology comparison of the amino acid sequences of *PKD1* between homo sapiens and other animals is shown in Supplementary Figures 2A,B. The mutated amino acid variant was located in the conserved region, indicating that the mutation might affect the gene's protein function. Cases III-2 and III-7 had a history of PKD but did not undergo genetic screening as they were deceased. Sanger sequencing identified a mutation of the *PKD1* gene (c.8360G>C p.R2787P) in cases IV-8, V-5, and V-8. Abdominal DU showed multiple renal and liver cysts (Supplementary Figure 1). The detailed characteristics of these patients with PKD are outlined in Table 2. This variant was thus co-segregated in the family and considered a likely pathogenic variant (PM1+PM2_Supporting+PP1+PP2+PP3) according to the ACMG guidelines.

**Table 2:** Clinical and genetic features of six ADPKD patients.

**Patient**	**Sex**	**Age** **(years)**	**First discovery (age, years)**	**ESKD at age (years)**	**Deceased at age (years)**	**Creatinine (umol/L)**	**Kidney manifestation**	**Extra-renal manifestation**	**Imaging**	**PKD1**
III-2	F	NA	40	40	49	NA	NA	NA	NA	NA
III-7	M	NA	40	50	69	NA	NA	NA	NA	NA
IV-3	M	70	40	60	Alive	1,176	hypertension, hematuria	Polycystic liver	MRI: polycystic kidney and polycystic liver	p.R2787P
IV-8	F	57	40	Normal	Alive	Normal	hypertension, urinary tract infection, urinary stones	Polycystic liver	DU: polycystic kidney and polycystic liver	p.R2787P
V-5	M	44	25	Normal	Alive	Normal	hypertension, urinary stones	Polycystic liver	DU: polycystic kidney and polycystic liver	p.R2787P
V-8	M	32	32	Normal	Alive	Normal	urinary stones	Polycystic liver	DU: polycystic kidney and polycystic liver	p.R2787P

NA, information not available; MRI, abdominal magnetic resonance imaging; DU, abdominal Doppler ultrasound; p.R2787P, carried the PKD1 p.R2787P mutation by genetic testing.

## Discussion

The present study has described a large Chinese family with 10 patients with ALS phenotype-−3 with asymptomatic *SOD1* p.H47R variant carriers and 6 with the ADPKD phenotype—in whom we performed WES, MLPA, and Sanger sequencing. The ADPKD phenotype was caused by a novel, likely pathogenic mutation (c.8360G>C, p.R2787P) in *PKD1*, and the ALS phenotype was caused by a pathogenic variant (c.140A>G, p.H47R) in *SOD1*. This is the first reported family to have simultaneously co-occurring ADPKD and ALS.

*SOD1*, which is on chromosome 21q22.11 and encodes Cu/Zn superoxide dismutase, was identified as the first ALS causative gene in 1993 (13). The SOD1 protein acts as an antioxidant enzyme by converting two superoxide anions, which are byproducts of cellular respiration with oxidant action, into hydrogen peroxide and oxygen (14). Mutant *SOD1* induces numerous toxic effects associated with pathological misfolding and aggregation of mutant *SOD1* species. To date, more than 238 *SOD1* mutations have been reported (http://www.hgmd.cf.ac/uk/). In our previous research, we found that *SOD1* is the most common causative gene, followed by *FUS, TARDBP, VCP*, and *OPTN* and that it accounts for 21.88% of fALS in Southwest China (15, 16). Analyzing this large family indicated that the patients with ALS carried *SOD1* p.H47R variant (also coded as p.H46R), which has been reported to be the most frequent variant in Chinese ALS patients with *SOD1* mutations (17). All our patients with the *SOD1* p.H47R variant presented with a predominated lower motor neuron deficit phenotype, rare bulbar or cognitive involvement, and considerably slow progression with longer survival time, similar to previous reports of patients with ALS with the same *SOD1* mutation (18, 19). Thus, molecular diagnostics may be needed to distinguish this phenotype of ALS from pure lower motor neuron syndrome, such as SMA.

Herein, only the proband underwent CSF NFL assessment, which was significantly increased, consistent with previous reports (20, 21). Of particular note, reduced levels of toxic variants appear to be a promising therapeutic strategy for *SOD1-*related ALS (14). Further investigations of NFL changes and their relation to ALS progression in asymptomatic carriers are needed and will provide potentially significant future directions for research.

In this family, both IV-1 and his grandmother (II-2) suffered from ALS, while his mother (III-2) suffered from ADPKD. Therefore, we conclude that III-2 was a likely carrier of the *SOD1* variant, though she died of ESKD at age 49, likely prior to ALS symptom onset. III-4 was also a likely carrier of the pathogenic *SOD1* variant, as III-7 is affected by ADPKD. III-13 recalled that her father (II-3) suffered from transient hematuria at age 50, had no further consultation or diagnosis, and died of suicide at age 55. We thus suspect that the *PKD1* variant was inherited by II-3 and not by II-2 (co-occurrence in the same patient). However, one study limitation is that detailed PKD information from the offspring and other family members of II-3 could not be obtained since they had either lost contact or died. Short tandem repeat (STR) analysis was not performed since many third-generation family members had died and blood samples were unavailable.

ADPKD, the fourth leading cause of ESKD worldwide, remains untreatable (22). It is characterized by the development of fluid-filled renal cysts, causing progressive loss of kidney function and culminating in the need for renal replacement therapy or kidney transplant (23). The typical clinical phenotypes also include extrarenal manifestation with polycystic liver and arachnoid cysts. *PKD1* and *PKD2* account for ~85 and 15% of patients with ADPKD, respectively (24). *PKD1* encodes polycystin1, which contains a large N-terminal extracellular region, multiple transmembrane domains, and a cytoplasmic C-tail (25), and plays a role in renal tubular development. To date, more than 2,691 *PKD1* variants have been reported (http://www.hgmd.cf.ac.uk/). Herein, a novel heterozygous missense variant c.8360G>C (p.R2787P) in the *PKD1* was identified among the family members with ADPKD. These patients also presented with typical ADPKD phenotypes, consistent with previous reports (7).

To our knowledge, this is the first report of the coexistence of important hereditary diseases, ADPKD and ALS, in the same family. Notably, the occurrence of these disorders in the same individual may complicate their clinical presentation or lead to diagnostic delay. Although no member of this family had both diseases, possibly due to the rarity of the two genes on different chromosomes being transmitted simultaneously, this possibility cannot be completely eliminated. The study limitation is that detailed PKD information could not be obtained from other members of II-3's family, though we will continue to follow this family. Genetic counseling for members of such families should consider the overall family context.

## Data availability statement

The original contributions presented in the study are included in the article/Supplementary material, further inquiries can be directed to the corresponding author.

## Ethics statement

The studies involving human participants were reviewed and approved by the Ethics Committee of West China Hospital, Sichuan University. The patients/participants provided their written informed consent to participate in this study. Written informed consent was obtained from the individual(s) for the publication of any potentially identifiable images or data included in this article.

## Author contributions

HS designed and conceptualized the study. SL, JL, CL, YC, BC, TY, QW, BZ, XC, and HS contributed patient material and clinical data. SL wrote the first draft of the manuscript. HS, JL, and CL revised the manuscript. All authors contributed to the article and approved the submitted version.

## Funding

This study was funded by the Sichuan Science and Technology Program (Grant No. 2022ZDZX0023).

## Conflict of interest

The authors declare that the research was conducted in the absence of any commercial or financial relationships that could be construed as a potential conflict of interest.

## Publisher's note

All claims expressed in this article are solely those of the authors and do not necessarily represent those of their affiliated organizations, or those of the publisher, the editors and the reviewers. Any product that may be evaluated in this article, or claim that may be made by its manufacturer, is not guaranteed or endorsed by the publisher.
