# Supplementary material for: Clinical and genetic study of a Chinese family affected by both amyotrophic lateral sclerosis and autosomal dominant polycystic kidney disease
Source: Front Neurol. 2022 Oct 20;13:1004909. doi: 10.3389/fneur.2022.1004909 (PMC9630937; doi:10.3389/fneur.2022.1004909)
Supplement: Supplementary file 1 [file Data_Sheet_1.doc]

**Supplementary Figure 1: DU images of the case IV-8 associated with polycystic kidney disease**

**
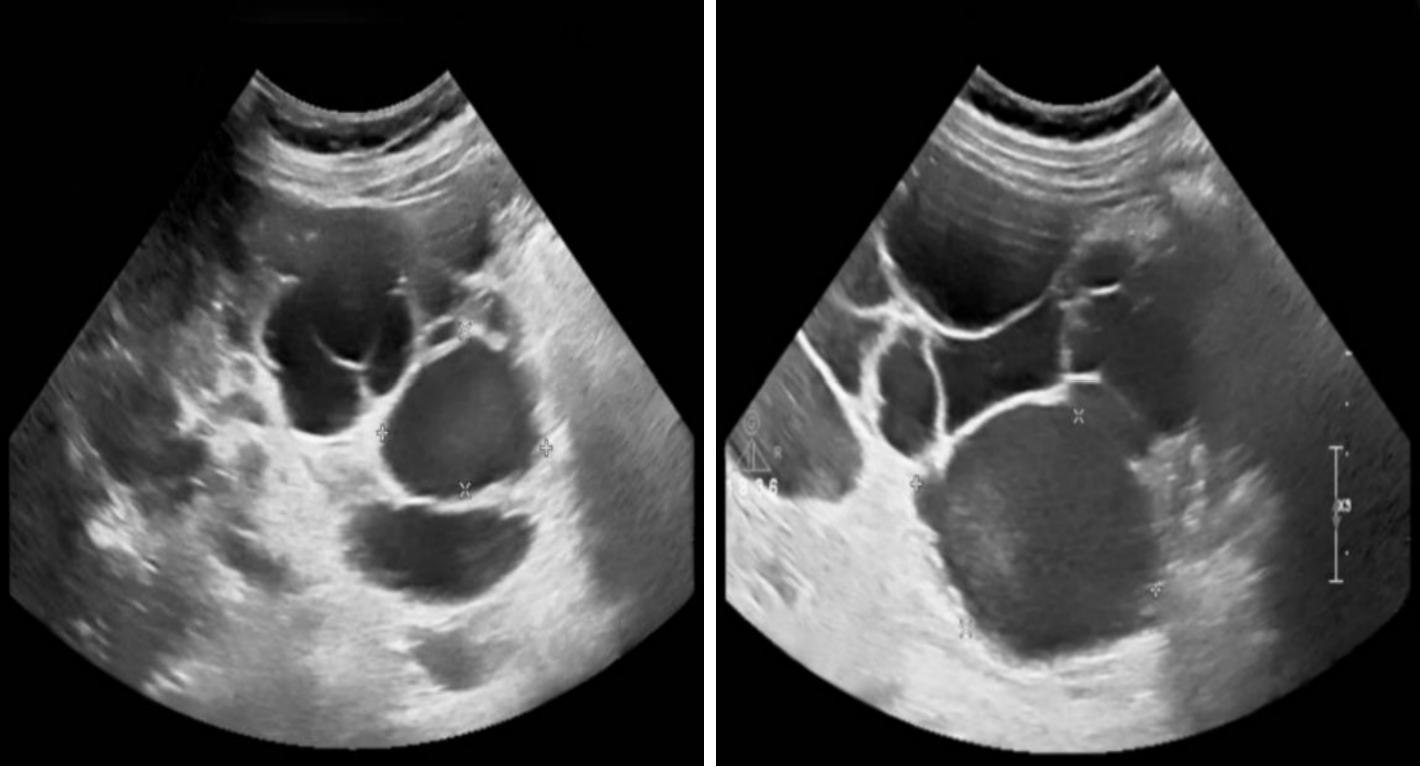
**

DU: doppler ultrasound of abdomen

**Supplementary Figure 2: Conservation analysis of PKD1**

(A) PKD1 c.8360G>C (p.R2787P) conservation analysis of species.


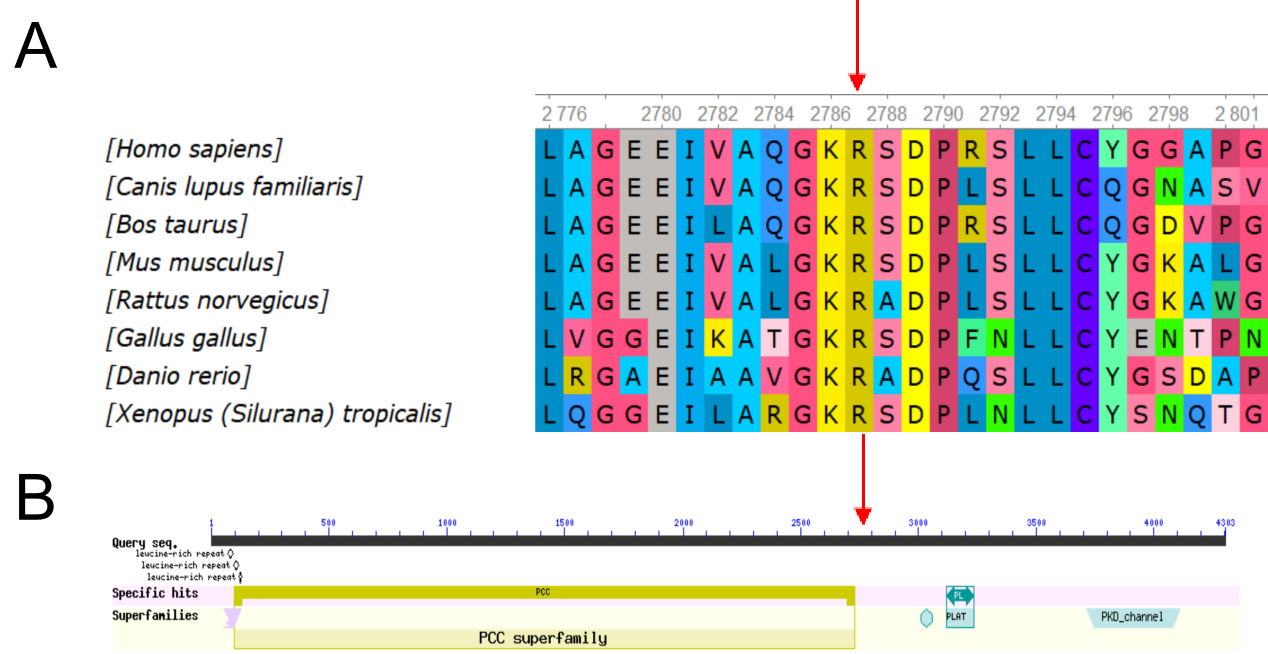
(B) PKD1 c.8360G>C (p.R2787P) conservation domains.

**Supplementary Figure 3: The PKD1 mutation is located in a functional protein domain**

(A) PKD1 c.8360G>C (p.R2787P) mutation is located in a functional protein in the Uniprot database.

(B) PKD1 c.8360G>C (p.R2787P) mutation is located in a functional protein in the Decipher database.

**A**

**
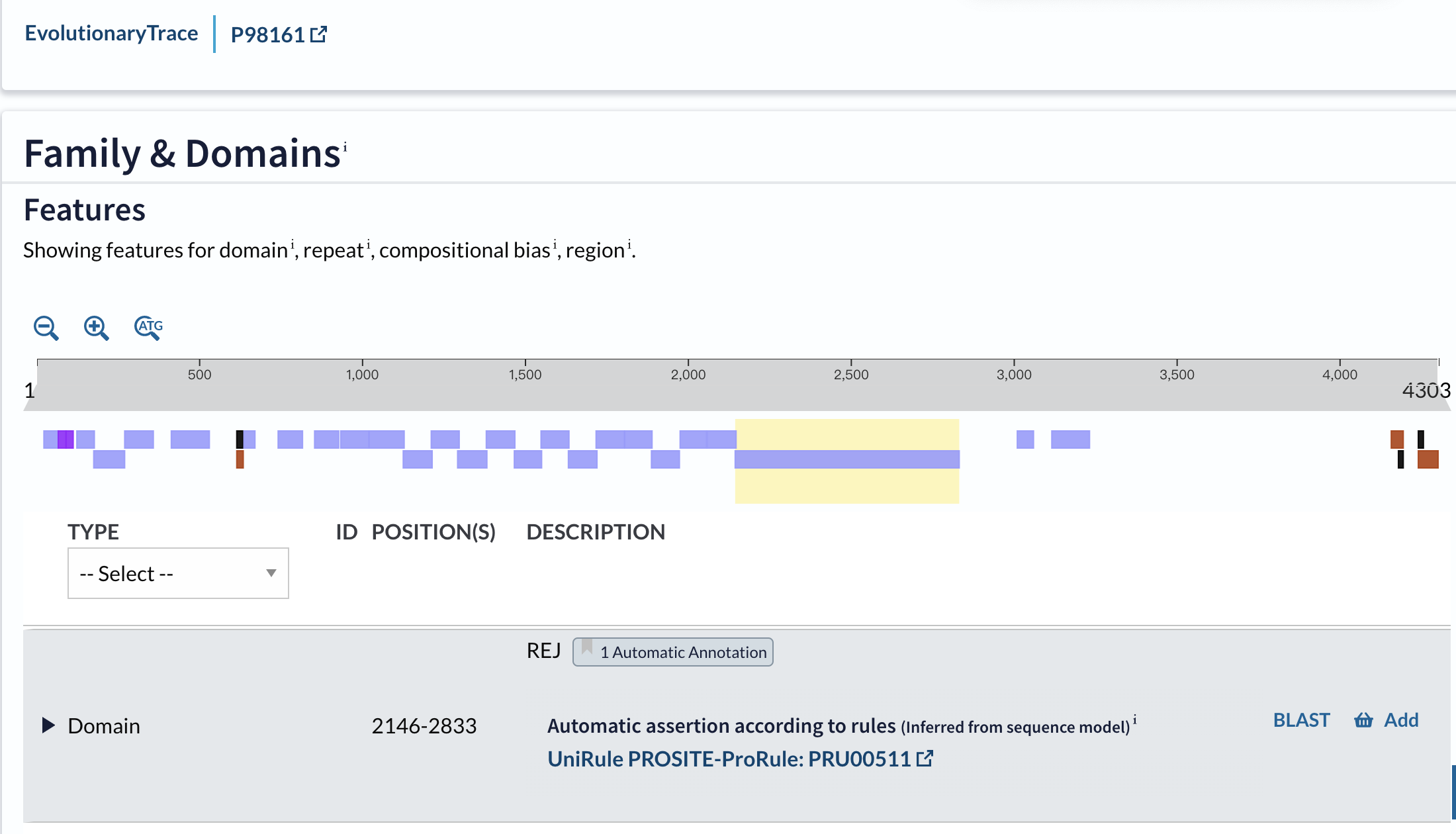
**

**B**

**
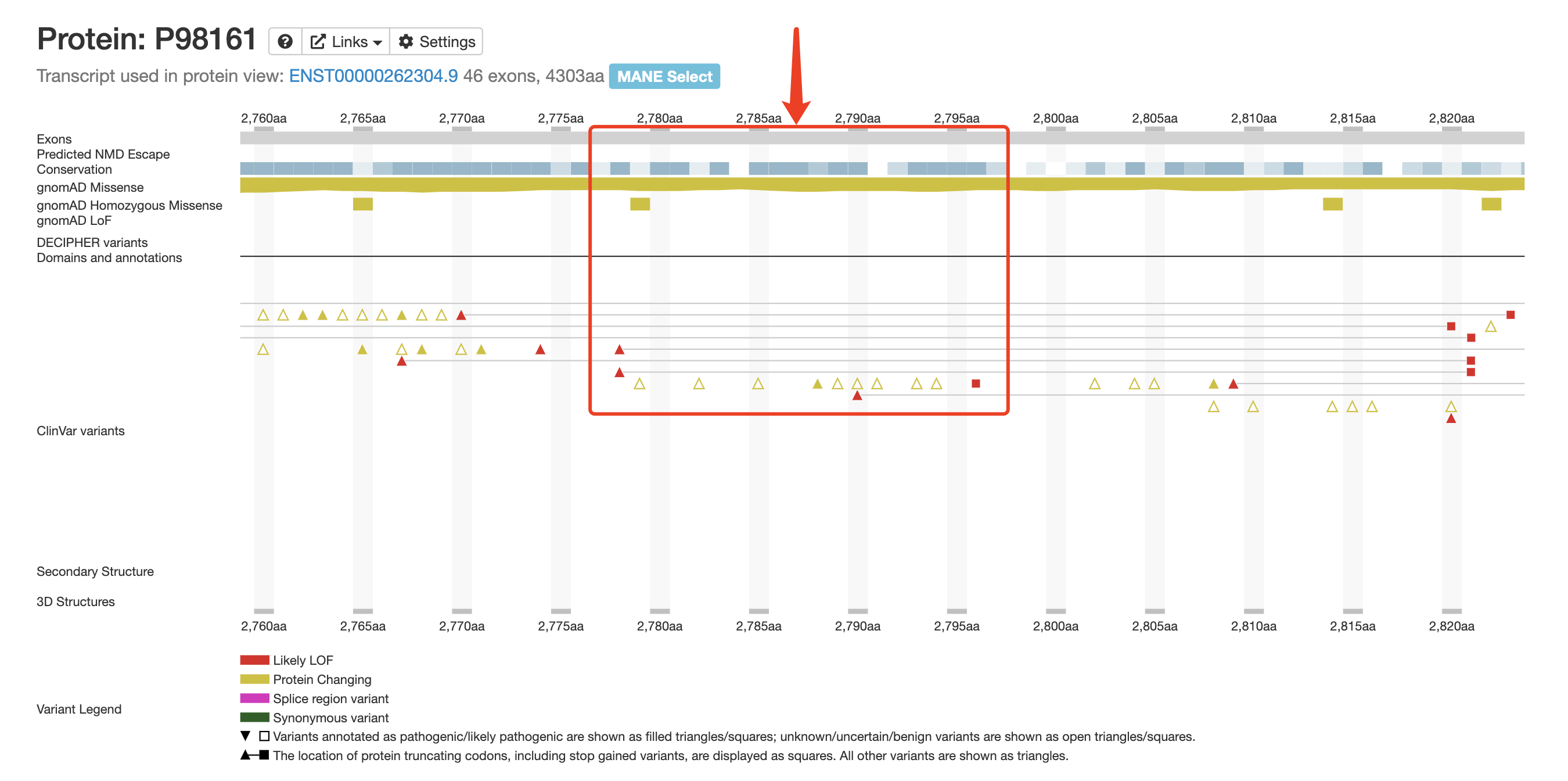
**

**Supplementary Figure 4: The IGV screenshot is displayed the number of reads supporting each allele.The sequencing depth of chr16:2153698 (PKD1 p.R2787P) is 438 and the “G” variant was 208.**

**
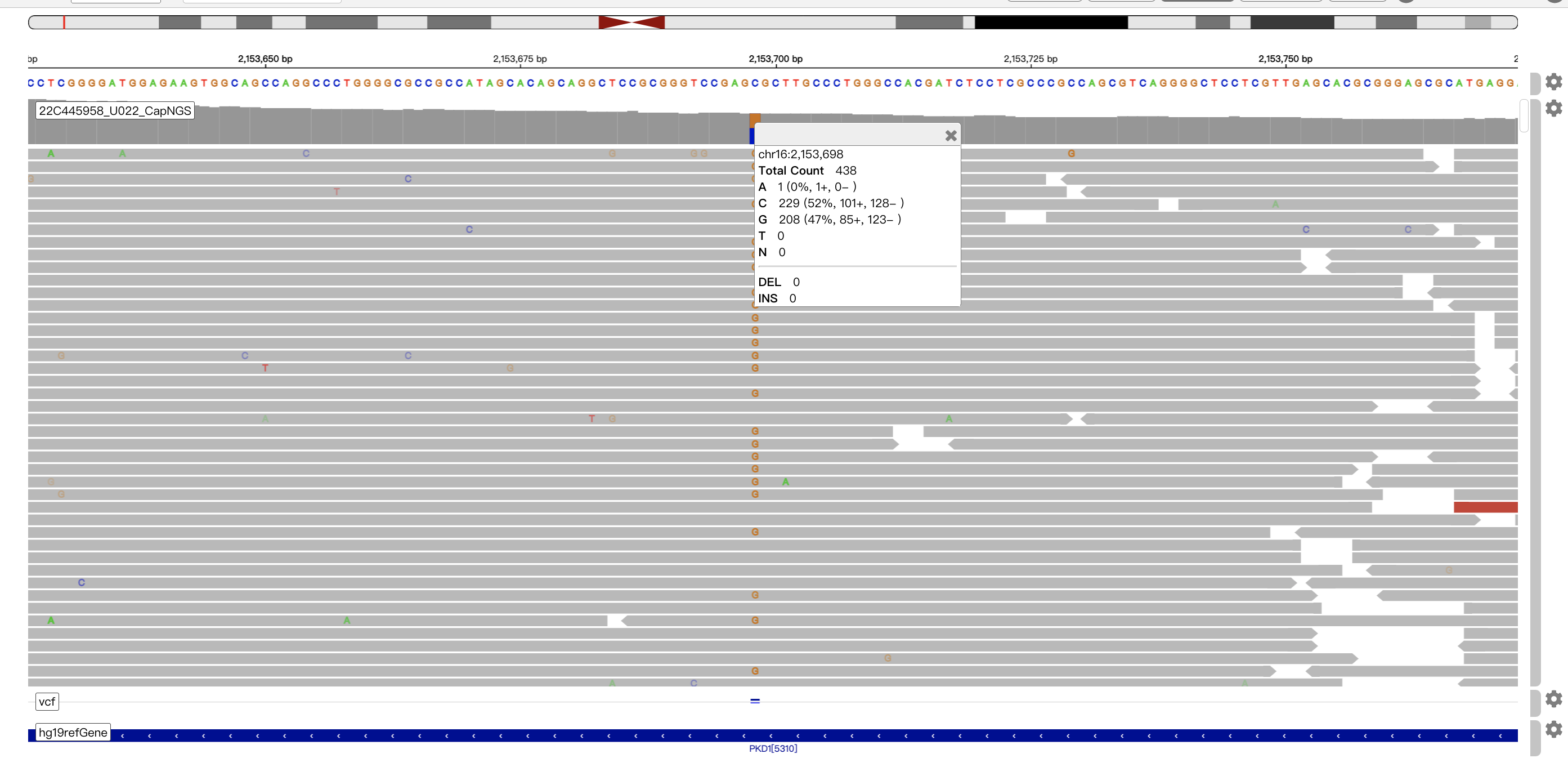
**

**Supplementary Table 1: Phenotype-genotype of the proband and the family members**

| Patient | Sex | Age  (years) | ALS phenotype | ADPKD phenotype | SOD1  (c.140A>G p.H47R) | PKD1  (c.8360G>C p.R2787P) | Other gene results |
| --- | --- | --- | --- | --- | --- | --- | --- |
| III-13 | F | 69 | + | - | SS:+ | SS:- | ND |
| IV-1 | M | 71 | + | - | SS:+ | SS:- | ND |
| IV-3 | M | 70 | _ | + | SS:- | WES:+ | MLPA: SMN1-, SMN2- |
| IV-8 | F | 57 | - | + | SS:- | SS:+ | ND |
| IV-11 | M | 64 | + | - | WES:+ | SS:- | ND |
| IV-13 | F | 58 | - | - | SS:- | SS:- | ND |
| IV-15 | F | 52 | + | - | WES:+ | SS:- | ND |
| IV-23 | F | 56 | + | - | SS:+ | SS:- | ND |
| IV-25 | F | 53 | + | - | SS:+ | SS:- | ND |
| IV-29 | F | 43 | - | - | SS:- | SS:- | ND |
| V-1 | M | 40 | - | - | SS:+ | SS:- | ND |
| V-3 | M | 19 | - | - | SS:+ | SS:- | ND |
| V-5 | M | 44 | - | + | SS:- | SS:+ | ND |
| V-7 | M | 37 | - | - | SS:- | SS:- | ND |
| V-8 | M | 32 | - | + | SS:- | SS:+ | ND |
| V-9 | F | 41 | - | - | SS:- | SS:- | ND |
| V-10 | M | 40 | - | - | SS:- | SS:- | ND |
| V-11 | M | 35 | - | - | SS:- | SS:- | ND |
| V-13 | M | 30 | - | - | SS:+ | SS:- | ND |

Sex: F:female; M:male; +: presence ; -:absence; WES:whole exome sequencing ; MLPA:multiplex ligation-dependent probe amplification ; SS：sanger sequencing; ND = not done.
